# Induction of VEGFA and Snail-1 by meningitic *Escherichia coli* mediates disruption of the blood-brain barrier

**DOI:** 10.18632/oncotarget.11696

**Published:** 2016-08-30

**Authors:** Ruicheng Yang, Wentong Liu, Ling Miao, Xiaopei Yang, Jiyang Fu, Beibei Dou, Aoling Cai, Xin Zong, Chen Tan, Huanchun Chen, Xiangru Wang

**Affiliations:** ^1^ State Key Laboratory of Agricultural Microbiology, College of Veterinary Medicine, Huazhong Agricultural University, Wuhan, Hubei 430070, China; ^2^ The Cooperative Innovation Center for Sustainable Pig Production, Huazhong Agricultural University, Wuhan, Hubei 430070, China; ^3^ Key Laboratory of development of veterinary diagnostic products of Ministry of Agriculture, Huazhong Agricultural University, Wuhan, Hubei 430070, China

**Keywords:** blood-brain barrier, tight junctions, vascular endothelial growth factor A, Snail-1, bacterial meningitis

## Abstract

*Escherichia coli* is the most common Gram-negative bacterium that possesses the ability to cause neonatal meningitis, which develops as circulating bacteria penetrate the blood-brain barrier (BBB). However, whether meningitic *E. coli* could induce disruption of the BBB and the underlying mechanisms are poorly understood. Our current work highlight for the first time the participation of VEGFA and Snail-1, as well as the potential mechanisms, in meningitic *E. coli* induced disruption of the BBB. Here, we characterized a meningitis-causing *E. coli* PCN033, and demonstrated that PCN033 invasion could increase the BBB permeability through downregulating and remodeling the tight junction proteins (TJ proteins). This process required the PCN033 infection-induced upregulation of VEGFA and Snail-1, which involves the activation of TLR2-MAPK-ERK1/2 signaling cascade. Moreover, production of proinflammatory cytokines and chemokines in response to infection also promoted the upregulation of VEGFA and Snail-1, therefore further mediating the BBB disruption. Our observations reported here directly support the involvement of VEGFA and Snail-1 in meningitic *E. coli* induced BBB disruption, and VEGFA and Snail-1 would therefore represent the essential host targets for future prevention of clinical *E. coli* meningitis.

## INTRODUCTION

Bacterial meningitis is the most important life-threatening infection of the central nervous system (CNS) with high morbidity and mortality, despite the advancements in antimicrobial treatment. It has been recognized as one of the top ten causes of infection-related death worldwide at present, and most survivors sustain neurological sequelae [1]. Most cases of bacterial meningitis initiate from hematogenous spread, and develop as circulating pathogenic bacteria penetrate the blood-brain barrier (BBB), destroy the brain parenchyma and thus cause CNS disorders [2]. The BBB consists of the brain microvascular endothelial cells (BMECs), pericytes and astrocyte [3, 4], which enables selective exchange of bioactive molecules between bloodstream and brain tissue, prevents circulating pathogens and toxins from entering the brain, thus maintains CNS homeostasis [5]. Pericytes are involved in regulation of vascular formation, stabilization and remodeling, therefore are responsible for maintaining the BBB properties [6]. BMECs, as the distinct and indispensable structural component of BBB, are characterized by the presence of tight junctions, which are dependent on the presence of several proteins such as Claudins, Occludin, β-catenin, as well as cytoplasmic zonala-occludin family members (e.g. ZO-1, -2, -3) [7, 8], which promote the high transendothelial electrical resistance and therefore impede paracellular transport of macromolecules [9]. Reducing TJ proteins expression and changes in distribution result in increased BBB permeability, which is an important indicator of BBB dysfunction [10].

There are actually quite a few bacteria possessing the ability to colonize the host brain and causing meningitis. Among these meningitic pathogens, *Escherichia coli* is the most common Gram-negative bacillary organism causing meningitis during the neonatal period [11], and this particular *E. coli* population is generally defined as neonatal meningitis *E. coli*, which belongs to the subgroups of extraintestinal pathogenic *E. coli* (ExPEC) [12]. Several lines of evidence from human cases and experimental animal models of *E. coli* meningitis indicate that meningitic *E. coli* strains exhibit the ability to invade BMECs *in vitro* and this invasion ability is well correlated with bacterial penetration into the brain *in vivo*. Thus, many early studies have mainly focused on the identification of potential *E. coli* determinants contributing to bacterial across the BBB, their interactions with host receptors, and the possible signaling pathway involved [13]. Notably, it is proposed that disruption of BBB junctions is an important event in some bacteria-mediated meningitis, usually results from the combined effect of bacterial invasion of BMECs, possible cellular injury by bacterial cytotoxins, and/or activation of host inflammatory pathways, which together compromise the barrier function and lead to CNS disorder [14]. However, whether meningitic *E. coli* induces the BBB disruption, and how does meningitic *E. coli* regulate this process are poorly understood.

Vascular endothelial growth factor A (VEGFA) is currently known as the most effective “activator” to increase the permeability of venule and postcapillary venule [15]. VEGFA can contribute to cell division, which regulates angiogenesis of the vascular endothelial cell [16]. Also, VEGFA can cause changes of the extracellular matrix [17], which plays certain roles in inflammation, wound healing, heart ischemia, atherosclerosis, tumor formation and many other pathological processes [18, 19]. A previous study has reported that VEGFA deriving from *E. coli*-infected BMECs can bind with VEGFR-1 in pericytes so as to negatively regulate survival of the pericytes [20]. So far, VEGFA and its receptor VEGFR have attracted great attention for their roles in tumor angiogenesis and anti-angiogenesis therapy [21], but their functions in meningitic *E. coli* infection remain little investigated.

Snail-1 is a zinc-finger transcription repressor that implicated in many physiological and pathological processes including normal embryonic development, repair of epithelial injury, as well as cancer metastasis [22–24]. Moreover, increasing studies have supported the involvement of Snail-1 in regulation of the TJ proteins, thus affecting the intercellular permeability [25]. Recent study has demonstrated that Group B *Streptococcus* (GBS) induction of Snail-1 impeded the expression of TJ proteins in human BMEC (hBMEC) [26]. However in *E. coli* meningitis, whether Snail-1 plays certain regulation on TJ proteins and mediates the BBB disruption are unclear, and the involving signaling pathways need to be addressed.

In this study, we provided evidences that meningitic *E. coli* invasion of hBMEC induced the upregulation of VEGFA and Snail-1 via TLR2-MAPK-ERK1/2 signaling pathway. Induction of VEGFA and Snail-1 decreased the expression of TJ proteins, resulting in the increase of the BBB permeability. Moreover, meningitic *E. coli* infection led to substantial production of proinflammatory cytokines and chemokines, which also promoted the upregulation of VEGFA and Snail-1, further accelerating the BBB disruption. These observations suggest an important mechanism for meningitic *E. coli* induced disruption of the BBB, in which VEGFA and Snail-1 serve as the key targets for meningitic *E. coli* mediated CNS damage.

## RESULTS

### *In vitro* and *in vivo* screening and characterization of meningitic *E. coli* strains

**Figure 1:**
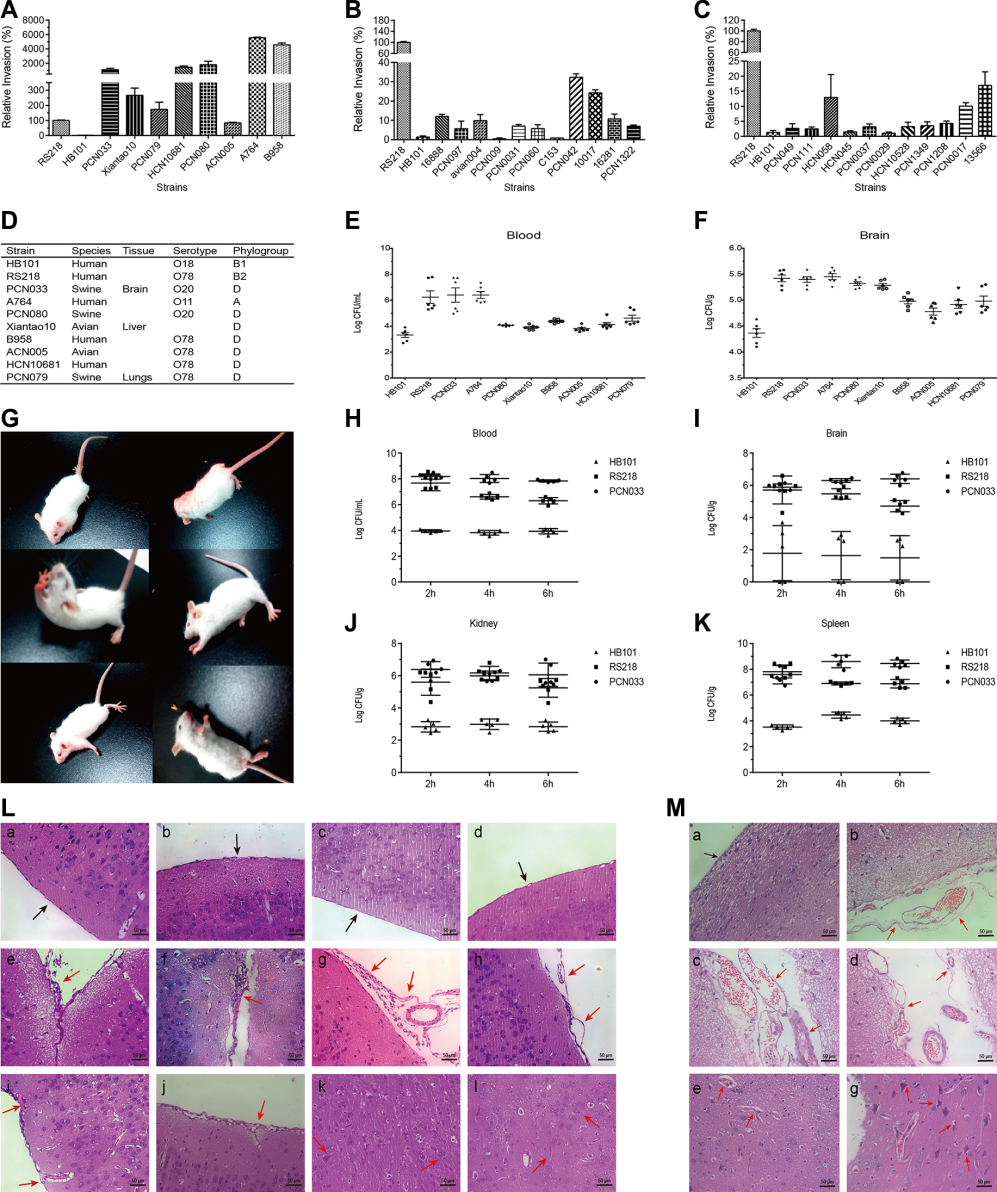
Screening and characterization of the meningitic *E. coli* isolate PCN033 (**A**–**C**) 30 ExPEC strains, including 7 strains from human patients, 3 strains from avian species, and 20 strains from pigs, were tested for their *in vitro* invasion of hBMEC. *E. coli* RS218 and HB101 were used as positive and negative controls, respectively. Results are presented as the relative invasion compared with that of RS218. (**D**) Brief background of the strains that chosen for the *in vivo* experiments in Figure E and F. (**E**–**F**) Survival in the blood (E) and invasion of the brain (F) were determined in 8 strains showing high invasion *in vitro*. Data are expressed as mean ± SD. (**G**) Typical neurological signs in mice receiving the challenge of meningitic *E. coli* PCN033, including overexcited, paddling, circling, trembling, and opisthotonus. (**H**–**K**) Mice were intravenously infected with PCN033 (*n* = 5), RS218 (*n* = 5), or HB101 (*n* = 5) at 1 × 10^7^ CFUs for 2, 4, and 6 h, at which time point the bacterial loads in the blood, brains, kidneys, and spleens were compared. Data are expressed as mean ± SD. (**L**) Brain histopathological changes in PCN033-challenged mice with neurological signs were examined by H&E staining. Panels a–d represent the control meninges from uninfected mice (black arrows). Panels e-l represent the brains from challenged mice, and red arrows indicate meninges disruption (e–h), hyperaemia (f, i), inflammatory cells accumulation (e–g, i, j), and embattled neurons (k, l). (**M**) Histopathological changes in brains from PCN033-challenged pigs. Panel a shows the normal meninges (black arrow). Panels b–f represent the typical pathological lesions with meninges thickening or separation (b–d), hyperaemia (b–f), and embattled neurons (f), as indicated by the red arrows.

We have collected and preserved lots of ExPEC strains with diverse backgrounds. To screen out the isolates with potential of causing CNS infection, we selected 30 strains, including 7 strains from patients, 3 strains from avian and 20 strains from diseased pigs, to testify their invasion abilities. The neonatal meningitis-causing *E. coli* RS218 and the non-meningitic *E. coli* HB101 were used as positive and negative controls, respectively. 8 strains were observed to possess strong invasion abilities, with similar or higher invasion compared with RS218 (Figure 1A–1C). We further tested these 8 strains (Figure 1D) for their abilities to invade the brain *in vivo*. Mice were intravenously challenged at the dosage of 1 × 10^7^ CFU for 2 h followed by cardiac perfusion. We found that the circulating bacterial load of PCN033 and A764 reached the similar level as that of RS218, while the others maintained a lower level as that of HB101, or slightly higher than HB101 (Figure 1E). In brains, these 8 strains exhibited varying degrees of invasion, and their bacterial loads were either significantly higher than that of HB101 or as higher as RS218 (Figure 1F). Two strains, PCN033 and A764, almost behaved the same way as the positive control RS218 in their survival in the blood and colonization of the brain, and thus were likely to be the potential meningitic *E. coli* isolates. Considering that PCN033 was originally isolated from the brain of diseased pig [27], we subsequently used PCN033 as our experimental meningitic strain, and further characterized its pathogenic phenotypes *in vivo*. Mice were challenged with PCN033 for multiple time points and the neurological signs, histopathological changes, as well as bacterial colonization, were recorded. As shown in Figure 1G, a series of neurological signs were observed in mice 6 h post infection and nearly all the mice showed severe symptoms or moribund, such as paddling, circling, trembling, and opisthotonus. Meanwhile, the *in vivo* bacterial survival and colonization were investigated after 2 h, 4 h, and 6 h of infection. The results showed that PCN033 maintained a high level of bacteremia as that of RS218 at each time point, which were significantly higher than that of HB101 group (Figure 1H). Accordingly, PCN033 and RS218 colonization in the brain, as well as in kidney and spleen, were also much higher than that of HB101 (Figure 1I–1K). These results indicated the strong ability of PCN033 in forming bacteremia and colonizing the brain, and the colonized bacteria could not be cleared efficiently. Moreover, the histopathological changes of the brains from the PCN033 challenged mice (Figure 1L), as well as piglets (Figure 1M), were observed including meninges thickening or separation, hyperaemia, inflammatory cell accumulation, and neuronophagia. Together, these findings confirmed that PCN033 was a meningitic *E. coli* strain with the ability to colonize the brain and cause CNS disorder.

### Meningitic *E. coli* PCN033 infection increased the BBB permeability *via* downregulating and disrupting the TJ proteins

**Figure 2:**
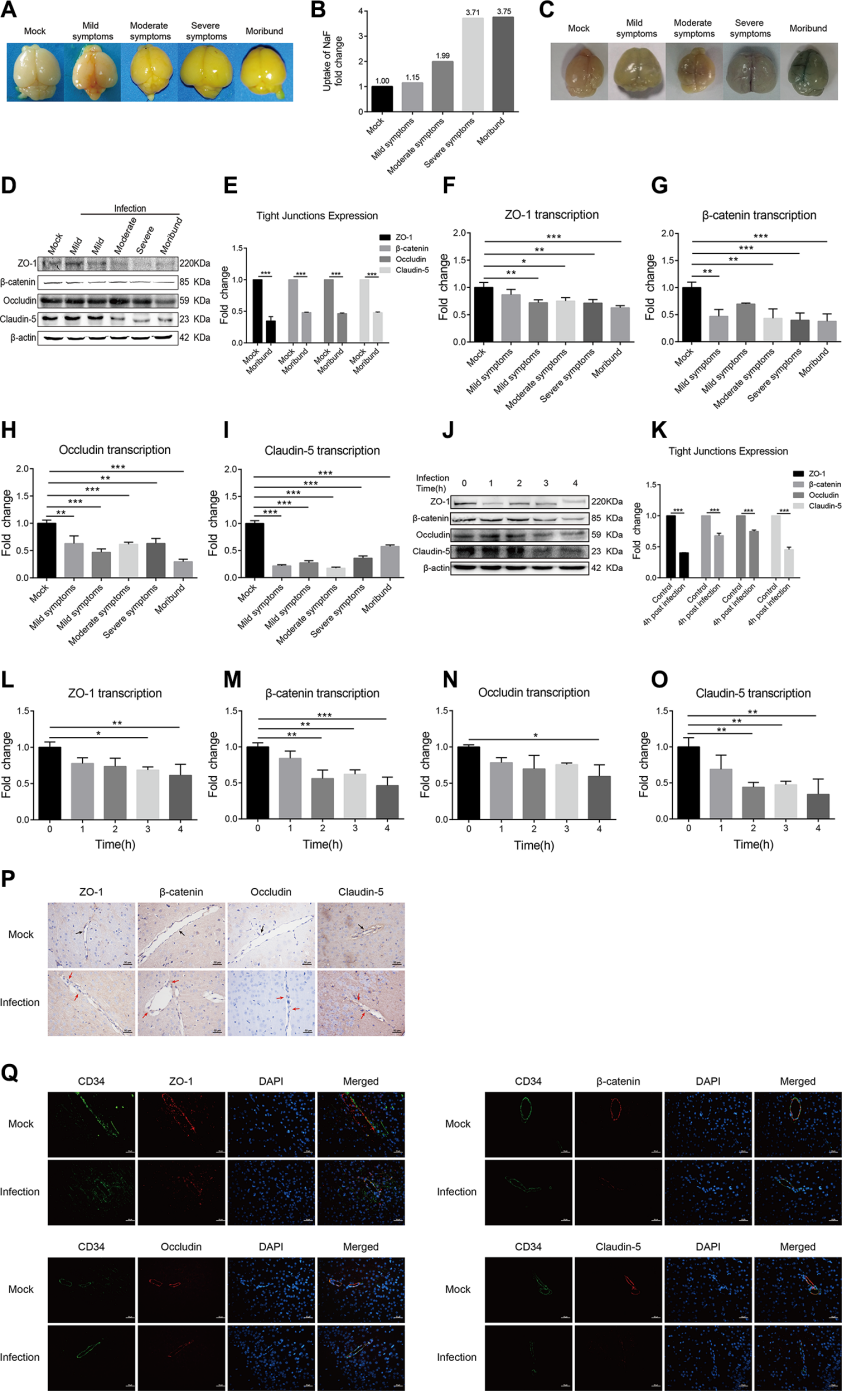
Meningitic *E. coli* PCN033 enhanced the permeability of BBB via inducing downregulation and disorganization of the TJ proteins (**A**–**B**) Mice were challenged by PCN033 until the appearance of different neurological signs, and NaF was injected intraperitoneally to allow diffusion. Uptake of NaF in the brain was analyzed by the fluorescence relative to that in the peripheral blood. (**C**) Evan's blue was injected to evaluate the integrity of the BBB. Because of the large molecular weight of Evan's blue by binding of serum albumin, only the increased permeability of the brain was observed in the moribund mice. (**D**–**E**) Brain lysates from the challenged mice were assessed via Western Blotting for the expression of TJ proteins, including ZO-1, β-catenin, Occludin, and Claudin-5. The β-actin was used as the loading control, and densitometry of the bands was performed to analyze the change of the protein expression upon infection. (**F**–**I**) Total RNA extracted from brains was analyzed by real-time PCR for the transcription of the TJ proteins. The β-actin gene was used as the internal reference for the brain RNAs. (**J**–**K**) Western blotting analysis of the TJ proteins in hBMEC in response to infection. β-actin was used as the loading control, and densitometry was performed to analyze the difference. (**L**–**O**) Real-time PCR analysis of the TJ proteins transcription in RNAs from infected hBMEC. GAPDH was used as the internal reference for the cellular RNAs *in vitro*. Analyzed data are presented as mean ± SD from three independent assays. (**P**–**Q**) Mice were challenged with or without PCN033 and brains from both groups of mice were analyzed for the integrity of vascular endothelium by IHC and IF. The TJ proteins ZO-1, β-catenin, Occludin, and Claudin-5 were selected as the markers reflecting the integrity of the vascular endothelium. In mock group, the TJ proteins were uniformly and closely arranged (Figure 2P, black arrows); in infected group, the arrangement of the TJ proteins became gapped, disrupted, and out-of-order (Figure 2P, red arrows). CD34 was specifically applied for labeling the vessel (Figure 2Q). Scale bar indicates 50 μm.

We next investigated if meningitic *E. coli* PCN033 could induce the disruption of the BBB. Small molecule dye sodium fluorescein (NaF, 376 Da), which can effectively permeate into the damaged tissues, was used to evaluate the alteration of BBB permeability after PCN033 challenge. Mice were infected by PCN033 and at different clinical stages (mild, moderate, severe and moribund), NaF extravasation was assessed. It is clear that NaF entry into the brain was higher in mice with more clinical signs (Figure 2A and 2B). To further confirm that BBB permeability is enhanced after infection, the infiltration of a larger molecule, Evan's blue (961 Da) was measured. As shown in Figure 2C, Evan's blue penetrated to the brain of the moribund mouse. Together, these observations directly evidenced that meningitic *E. coli* PCN033 infection can increase the BBB permeability. Since TJ proteins are the most important components of BBB which determine the paracellular permeability, we therefore determined the alteration of these TJ proteins in the brains of PCN033-infected mice by qPCR and Western Blotting. It was found that the expression of these junction-associated proteins were decreased, corresponding to the severity of the symptoms (Figure 2D). When mice became moribund, the TJ proteins were all significantly reduced (Figure 2E, *p <* 0.001). Meanwhile, the transcription of these TJ genes were also tested and exhibited the similar trends of downregulation (Figure 2F–2I). Since these junction-associated genes can be detected in hBMEC, the expression of these genes were monitored in the monolayers of hBMEC after infection. Similar results were obtained from the *in vitro* hBMEC model as that from the *in vivo* model, showing a decreasing of the TJ proteins (Figure 2J–2O). These *in vivo* and *in vitro* observations together supported the notion that meningitic *E. coli* infection downregulates the expression of the TJ proteins. In addition, immunohistochemistry (IHC) and immunofluorescence (IF) were performed to examine the distribution of these TJ proteins in the brain upon PCN033 infection. As shown in Figure 2P and 2Q, the TJ proteins (ZO-1, β-catenin, Occludin, and Claudin-5) were found to be well-organized and distributed around the brain vessels in the control mice. In contrast, the vascular endothelial layer became inconsecutively distributed, irregular or gapped in mice after PCN033 challenge, indicating a breakdown of the TJ proteins between adjacent endothelial cells. Therefore, these findings suggest that meningitic *E. coli* induces BBB disruption *via* downregulating the expression of the TJ proteins, and altering the distribution of these junctional proteins.

### Meningitic *E. coli* induction of VEGFA contributes to the increased BBB permeability

**Figure 3:**
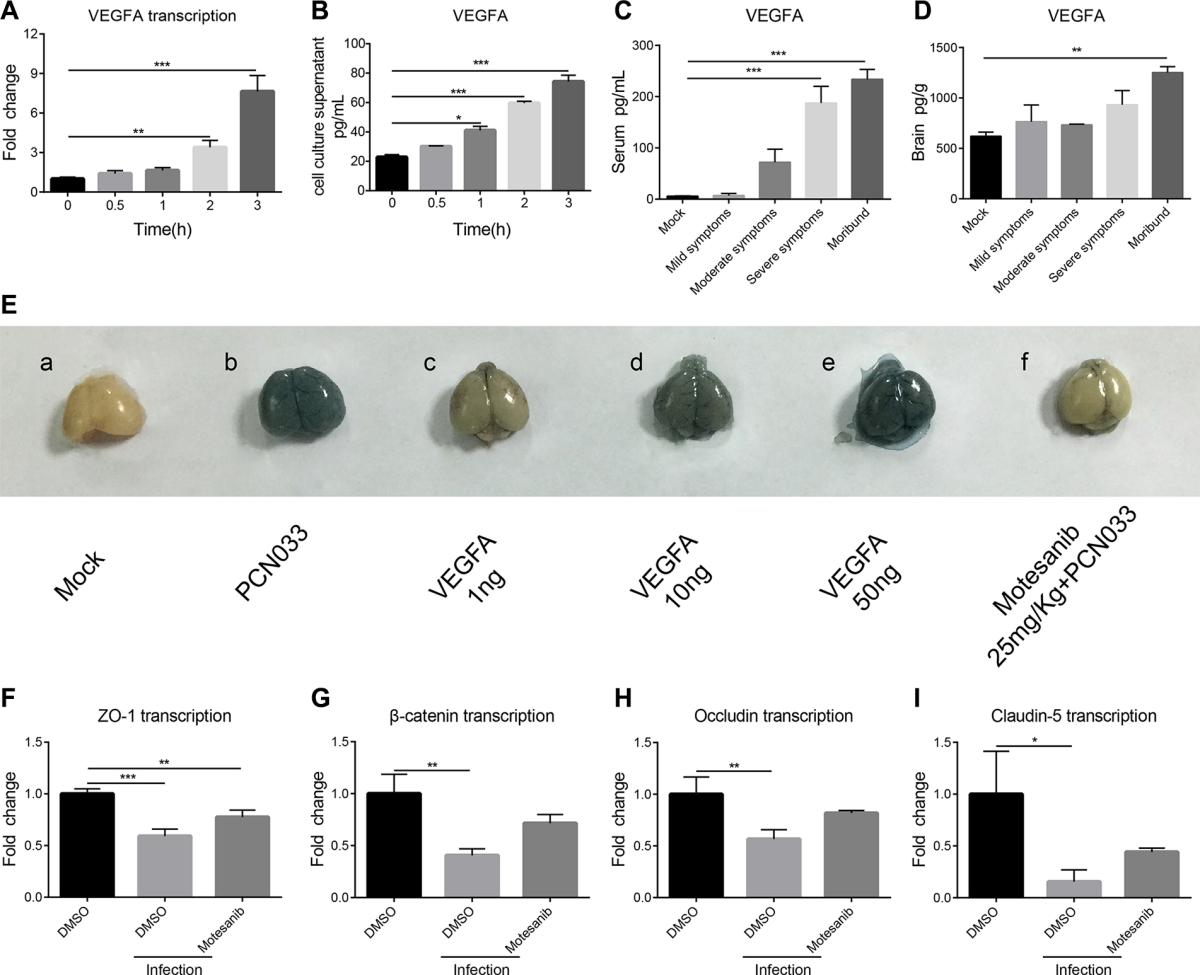
PCN033 induction of VEGFA mediated BBB permeability enhancement via downregulation of TJ proteins (**A**) Real-time PCR analysis of the VEGFA transcription in hBMEC in response to PCN033 infection at the MOI of 10. GAPDH was used as the internal reference for the quantitation. (**B**) Determination of the secretory VEGFA in the hBMEC culture supernatant by the ELISA kit. (**C**–**D**) ELISA analysis of the VEGFA amount in serum and brain lysates from the challenged mice exhibiting different symptoms. (**E**) Contribution of the VEGFA to the permeability of the BBB. VEGFA was injected into the mice at increasing doses and the BBB permeability was evaluated by Evan's blue. Motesanib diphosphate (25 mg/kg) was intraperitoneally administrated 2 h before PCN033 challenge, and the BBB permeability was tested by Evan's blue method. (**F**–**I**) Expression of the TJ proteins (ZO-1, β-catenin, Occludin and Claudin-5) in hBMEC upon infection with or without VEGFR inhibition. Data are expressed as mean ± SD from three independent experiments.

Since VEGFA plays important roles in cell growth, proliferation, permeability, and angiogenesis of the vascular endothelial cell [28], we next investigated if VEGFA is involved in meningitic *E. coli*-induced BBB disruption. It was found that the VEGFA transcription increased significantly 2 h post challenge, compared with that in the uninfected cells (Figure 3A). Likewise, VEGFA was found to be significantly increased in the culture supernatant in a time-dependent manner (Figure 3B). In mice challenged with PCN033, the circulatory VEGFA in the serum exhibited a sharp increase, and the amount of VEGFA in the serum from mice with severe symptoms and moribund were significantly higher than that in mice with mild or moderate symptoms, as well as uninfected mice (*p <* 0.001, Figure 3C). The VEGFA in the brain also exhibited an increase, with VEGFA from moribund mice significantly higher than other groups of mice (*p <* 0.01, Figure 3D). Since VEGFA exhibited a huge increase in the blood, we subsequently investigated whether this increased circulatory VEGFA has any effect on the BBB permeability. Indeed, intravenous (tail vein) injection of recombinant VEGFA resulted in a dose-dependent increase of the BBB permeability (Figure 3E). VEGFA functions through interaction with its receptor VEGFR, and we next blocked the VEGFA effects by the pretreatment of 25 mg/kg motesanib diphosphate, an inhibitor of VEGFR. It was found that blocking VEGFA function could significantly decrease meningitic *E. coli*-induced increasing of the BBB permeability (Figure 3E, group f vs group b). This provides direct evidence for the VEGFA-VEGFR interaction in influencing the permeability of the BBB. Furthermore, the effects of VEGFA on meningitic *E. coli* regulation of the TJ proteins were analyzed by using VEGFR inhibitor motesanib diphosphate. From the qPCR results, the meningitic *E. coli* infection significantly decreased the expression of the TJ proteins, consistent with our above observations, while pretreatment with the VEGFR inhibitor largely offset the downregulation of the TJ proteins by meningitic *E. coli* (Figure 3F–3I). These data suggest that blockage of VEGFA function effectively attenuated meningitic *E. coli* downregulation of the TJ proteins. We additionally observed that blocking VEGFA dose-dependently decreased PCN033 invasion of hBMEC (Supplementary Figure S1). In combination, the above data supported that meningitic *E. coli* infection could induce the upregulation of VEGFA, which promoted the increase of the BBB permeability.

### Meningitic *E. coli* induction of Snail-1 negatively regulates the TJ proteins expression

**Figure 4:**
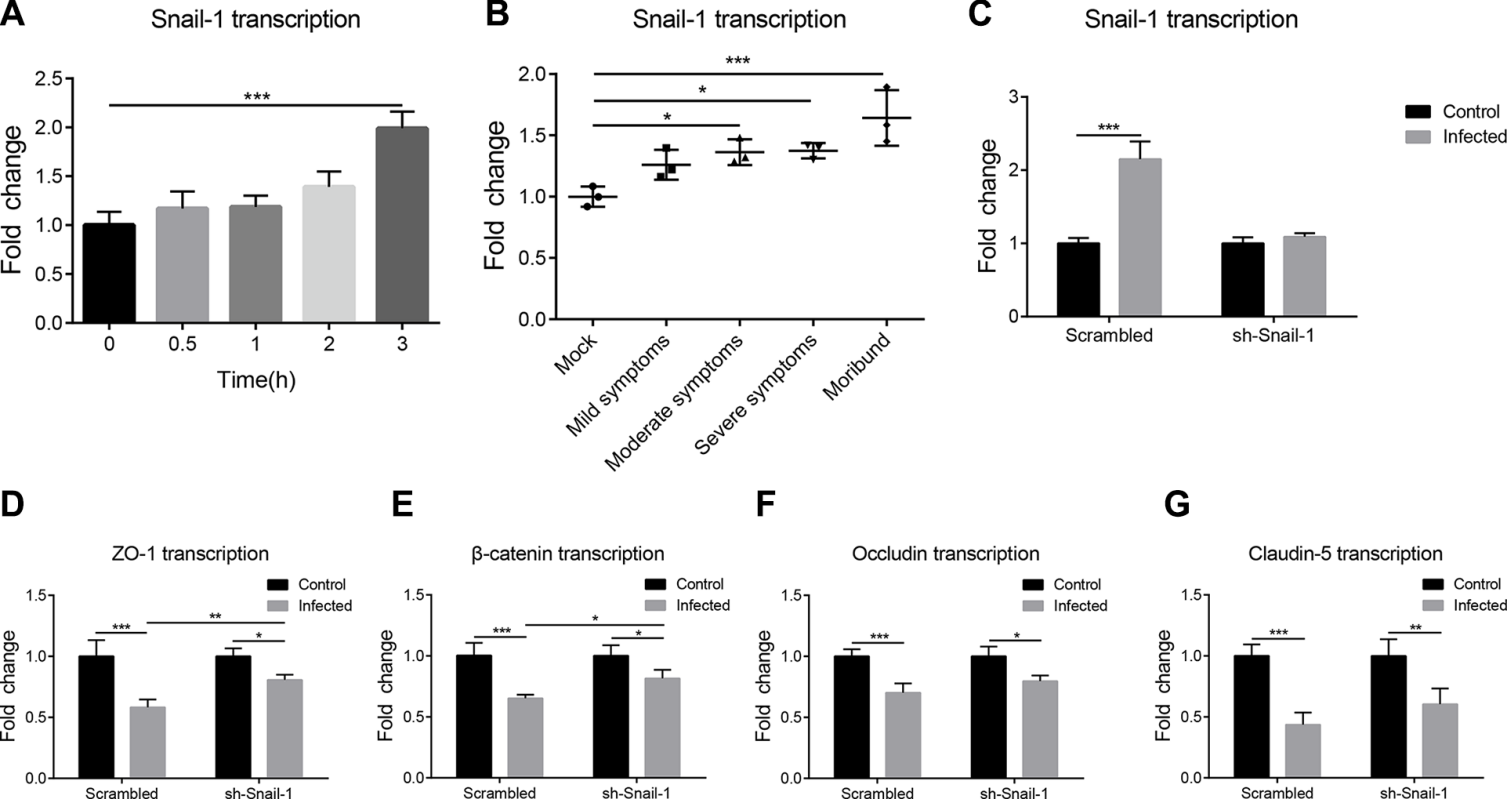
PCN033-induced upregulation of Snail-1 mediated the decrease in TJ proteins (**A**) Real-time PCR analysis of Snail-1 transcription in hBMEC in response to PCN033 challenge. GAPDH was used as the internal reference. (**B**) Snail-1 transcription was determined in total brain RNAs from infected mice exhibiting different signs. The transcription of β-actin was used as the internal reference. (**C**) PCN033-induced upregulation of Snail-1 in hBMEC was blocked by Snail-1 shRNA transfection, but not by the scrambled transfection. (**D**–**G**) Snail-1 knocking-down via shRNA partly offset the downregulation of TJ proteins by PCN033 infection compared with that in cells with scrambled transfection. Results are shown as mean ± SD from three independent assays.

Studies have indicated the importance of Snail-1 in regulation of the TJ proteins. Since meningitic *E. coli* infection induced the BBB disruption *via* downregulating TJ proteins, we investigated whether this TJ proteins downregulation was mediated by Snail-1. The Snail-1 transcription in either the infected hBMEC or the brains of challenged mice were determined by qPCR. On the hBMEC model, the Snail-1 transcription showed a time-dependent increase after infection (Figure 4A). In the challenged mice, the transcription level of Snail-1 in the brain also showed a gradual increase as the infection progressed, with significant increasing in the brains of mice showing moderate to severe symptoms, and even worse (Figure 4B). These together indicate the upregulation of Snail-1 in response to meningitic *E. coli* infections *in vitro* and *in vivo*. Subsequently, we made the Snail-1 knocking-down in hBMEC by shRNA. The Snail-1 upregulation in response to PCN033 was fully limited in the transfected hBMEC (Figure 4C). We further compared the regulation of Snail-1 on the TJ proteins in both sh-Snail-1 transfected cells and the scrambling-transfected cells in response to the infection. Although the downregulation of TJ proteins upon infection were not completely abolished, an attenuated decrease of the TJ proteins in the transfected hBMEC was observed when compared with those in the scrambling-transfected cells (Figure 4D–4G). These reflect that infection-induced Snail-1 could downregulate some of the TJ proteins, thus participating in the enhancement of the BBB permeability.

### Upregulation of VEGFA and Snail-1 requires the infection induced activation of TLR2-MAPK-ERK1/2 signaling

**Figure 5:**
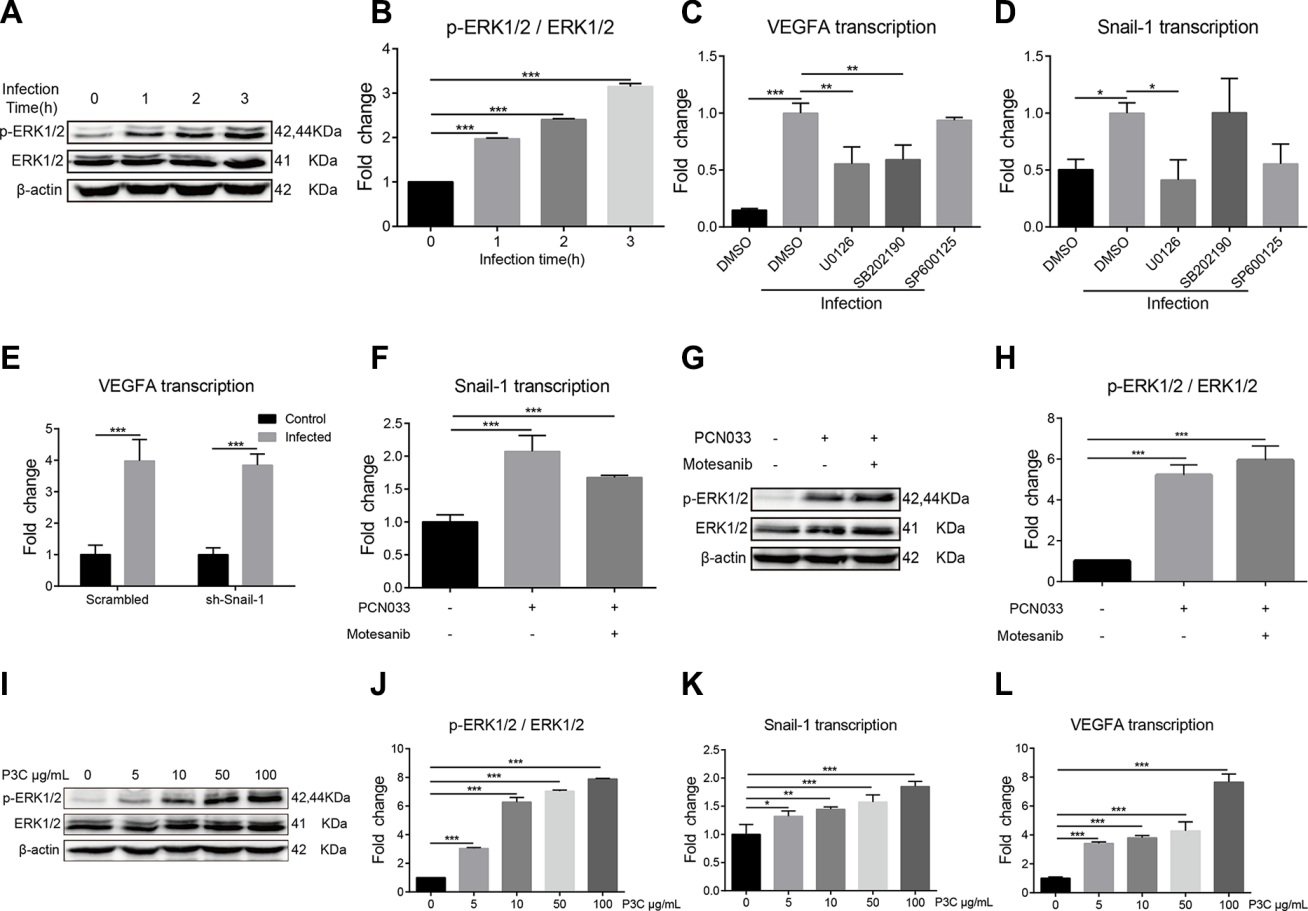
TLR2-MAPK-ERK1/2 signaling cascade is required for the induction of VEGFA and Snail-1 by PCN033 (**A**) Phosphorylation of ERK1/2 along with PCN033 infection. The β-actin was detected as the loading control. (**B**) Densitometrical analysis of the ERK1/2 activation in hBMEC 2 h post-infection, compared with that in uninfected cells. Data are calculated as the ratio of phospho-ERK1/2 to total ERK1/2. (**C**) Effects of the MAPK signaling inhibitors on the PCN033-induced upregulation of VEGFA. U0126 (selective inhibitor of ERK1/2) and SB202190 (selective inhibitor of p38) could significantly decrease the PCN033-induced upregulation of VEGFA, while SP600125 (specific inhibitor of JNK) could not. (**D**) Effects of the MAPK signaling inhibitors on the PCN033-induced upregulation of Snail-1. Selective ERK1/2 inhibitor U0126 could completely block the PCN033-induced upregulation of Snail-1. (**E**–**F**) Snail-1 knocking-down via shRNA in hBMEC did not affect the induction of VEGFA by PCN033, while blocking VEGFA pathway significantly decreased the upregulation of Snail-1. (**G**–**H**) Effects of the VEGFR inhibitors on PCN033-induced activation of ERK1/2 and the densitometric analysis. (**I**–**J**) TLR2 agonist Pam3CSK4 induced the activation of ERK1/2 in a dose-dependent manner. (**K**–**L**) Pam3CSK4 dose-dependently induced the upregulation of Snail-1 and VEGFA in hBMEC. Results are expressed as mean ± SD from three independent assays.

MAPK-ERK1/2 signaling pathway has been reported to play multiple roles in *E. coli* infection [29, 30], and recent studies have indicated the direct effect of MAPK-ERK1/2 phosphorylation on the regulation of Snail-1 [26]. We investigated if induction of VEGFA and Snail-1 in response to meningitic *E. coli* requires the activation of MAPK-ERK1/2 signaling. Firstly, we observed a time-dependent activation of ERK1/2 in response to the infection, with increased phosphorylation of ERK1/2 after *E. coli* infection of hBMEC (Figure 5A and 5B). By the treatment of different MAPK pathway inhibitors including U0126 (a specific inhibitor of ERK1/2), SB202190 (a specific inhibitor of p38) and SP600125 (a specific inhibitor of JNK), we subsequently found that both U0126 and SB202190 significantly decreased PCN033-induced upregulation of VEGFA (Figure 5C), and U0126 blocked the upregulation of Snail-1 (Figure 5D). The above observations suggest the common role of ERK1/2 in mediating the upregulation of both VEGFA and Snail-1 by meningitic *E. coli*. To investigate the possible interrelationship between VEGFA and Snail-1, the VEGFA transcription in the sh-Snail-1 transfected cells was compared to that in the scrambling-transfected cells upon infection. It was found that Snail-1 knocking-down did not affect bacterial induction of VEGFA (Figure 5E). Similarly, the blocking of VEGFA pathway could not prevent the significant upregulation of Snail-1 by infection (Figure 5F). Moreover, both VEGFA and Snail-1 were further supported to be downstream of MAPK-ERK1/2, by the demonstrations that blocking of VEGFA did not impede the phosphorylation of ERK1/2 (Figure 5G and 5H), and similarly the Snail-1 knocking-down did not affect bacterial induced activation of ERK1/2 (Data not shown).

TLR-signaling has been widely associated with diverse bacterial-derived stimulations, which usually requires the involvement of MAPK signaling [31]. As we have demonstrated the involvement of MAPK-ERK1/2 signaling in meningitic *E. coli* induced upregulation of VEGFA and Snail-1, whether the TLR triggers this signaling event in response to infection was analyzed. By using different TLR-signaling inhibitors or agonists, we observed that Pam3CSK4 (shortened as P3C, an agonist of TLR2) could dose-dependently stimulate the phosphorylation of ERK1/2 (Figure 5I and 5J), supporting the involvement of TLR2 in MAPK-ERK1/2 signaling in response to meningitic *E. coli*. Further, we found that P3C only could significantly induce the transcription of Snail-1 and VEGFA in a dose-dependent manner (Figure 5K and 5L), suggesting that TLR2 may act as a first responder leading to the activation of MAPK-ERK1/2 signaling. Therefore, these data indicate that meningitic *E. coli*-induced activation of TLR2-MAPK-ERK1/2 signaling is involved in the induction of VEGFA and Snail-1 in hBMEC.

### Meningitic *E. coli*-induced cytokines and chemokines promote upregulation of VEGFA and Snail-1

**Figure 6:**
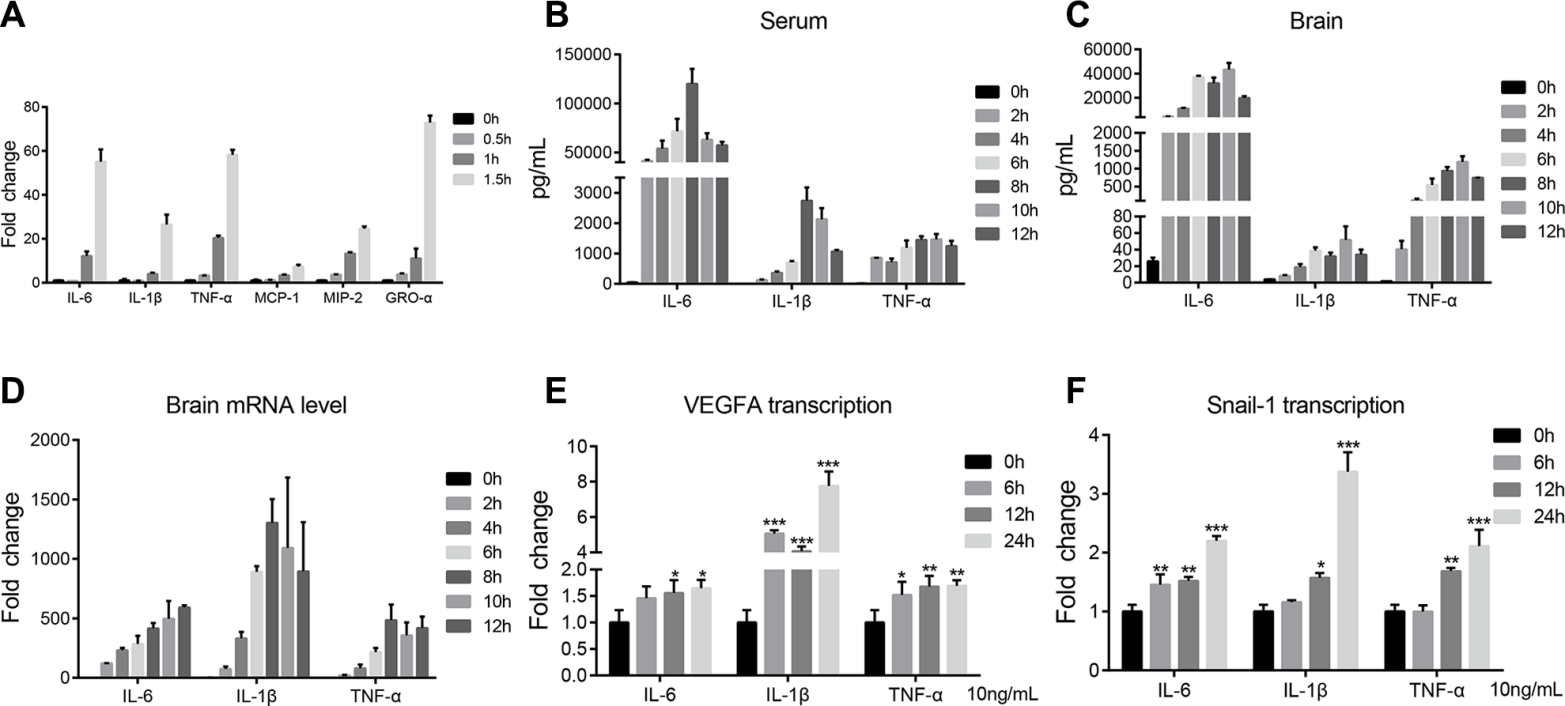
PCN033-induced production of cytokines and chemokines conduced to upregulation of VEGFA and Snail-1 (**A**) PCN033 infection of the hBMEC induced the upregulation of multiple cytokines and chemokines in a time-dependent manner, including IL-6, IL-1β, TNF-α, MCP-1, MIP-2, and GRO-α. (**B**–**C**) Multiplex analysis of the cytokines and chemokines in the serum and brains from challenged mice along with time. Partial cytokines results are presented here, which show significantly increased generation compared with uninfected mice. (**D**) QuantiGene expression profiling of the cytokines in brain RNAs from challenged mice. The representative cytokines, IL-6, IL-1β, and TNF-α, showed the time-dependent increase along with infection. (**E**–**F**) Stimulation of the cytokines (IL-6, IL-1β or TNF-α) on hBMEC at 10 ng/mL led to the upregulation of VEGFA and Snail-1 in a time-dependent manner. Data are means ± SD of results from independent experiments.

Pathogens-induced cytokines and chemokines have also been recognized as contributors to CNS damage in various models of neuroinflammation. Here, we investigated the induction of cytokines and chemokines by meningitic *E. coli*, and found that bacterial infection could time-dependently induce the upregulation of multiple cytokines (e.g. IL-6, IL-1β, and TNF-α) and chemokines (e.g. MCP-1, MIP-2, and GRO-α) (Figure 6A), while this induction was much lower in response to HB101 (Supplementary Figure S2). *In vivo*, by the approach of multiplex cytokines detecting and QuantiGene expression profiling, we also observed tempestuously production of these cytokines in the serum and brain lysates of PCN033-challenged mice (Figure 6B and 6C), as well as the mRNA transcription of these cytokines from the brain total RNA (Figure 6D). These indicate an intense inflammation in both the periphery and the CNS. More importantly, with treatment of 10 ng/mL cytokine (IL-6, IL-1β, and TNF-α), we observed a time-dependent increase in transcription of both VEGFA and Snail-1 in hBMEC (Figure 6E and 6F), with the greatest increase by the treatment of IL-1β. These findings suggest that meningitic *E. coli*-induced proinflammatory cytokines and chemokines could facilitate the upregulation of the VEGFA and Snail-1, two important host targets that mediate the BBB disruption.

## DISCUSSION

Different meningitis-causing microbes have developed different strategies for successful colonization and entry of the host brain [1]. The initiation of bacterial meningitis occurs when the circulating bacteria penetrate the BBB, develops as bacterial induction of BBB disruption, and finally leading to the CNS disorder. The TJ proteins, constituting the most important junctional structure of BBB, promote the cell polarity and barrier integrity, and maintain the stability of the CNS microenvironment [32–34]. Several studies have reported that meningitic pathogens could degrade TJ proteins or remodel the junctional complexes. For example, recent studies have supported that *Neisseria meningitidis* induced specific cleavage of Occludin through the release of matrix metalloproteinases 8 (MMP-8) [35]. Type IV pili of *N. meningitidis* mediated recruitment of the PAR3/PAR6/PKCζ polarity complex and delocalization of junctional proteins [36, 37]. GBS has been shown to disrupt TJ proteins in brain endothelium via utilizing host transcription factor Snail-1 [26]. In addition, early study has demonstrated that *E. coli* K1 strain OmpA-mediated adhesion of hBMEC induced PKCα signaling process that led to dissociation of β-catenin from Cadherins, thus resulting in disruption of the barrier integrity [38]. In our study, we further demonstrate that meningitic *E. coli* invasion of the brain could enhance the BBB permeability by downregulating the TJ proteins expression, and the TLR2-MAPK-ERK1/2 signaling-mediated induction of VEGFA and Snail-1 mainly contribute to this process. Meanwhile, the infection-induced generation of proinflammatory cytokines and chemokines promote the increase of VEGFA and Snail-1, thus also aggravating the damage of the TJ proteins.

Here, we confirmed that the brain isolate PCN033 was a potential meningitic *E. coli* strain by observations of the typical neurological symptoms in the challenged mice, as well as the histopathological changes of the brain. Previous studies largely supported the main involvement of *E. coli* K1 strain in *E. coli* meningitis, without any mention of *E. coli* meningitis caused by other non-K1 strains [1, 2]. However noticeably, our early whole genome sequencing and annotation indicated that PCN033 was a K2 capsule strain [27], and we herein demonstrated that both *E. coli* K1 strain RS218 and K2 strain PCN033 could survive in the blood and cross the brain, as well as induce the upregulation of VEGFA and Snail-1 in hBMEC via MAPK-ERK1/2 signaling (Figures 1, 5C, 5D, and Supplementary Figure S3). These suggest that *E. coli* K2 strain PCN033 might share certain characteristics as the K1 strain in the process of causing meningitis, and more work are required to further distinguish the differences of bacterial meningitis caused by *E. coli* K1 and K2 strains.

We provided direct evidences that PCN033 infection could increase the BBB permeability by using two different dyes, NaF and Evan's Blue. The extravasation of NaF was easily to be observed in the brains of challenged mice in an infection-dependent manner. In Evan's Blue assay, due to the larger molecular weight resulting from binding of the albumin, we only observed the obvious penetration of the dye into the brain from moribund mice, but not from mice with mild or moderate signs. These *in vivo* observations together indicate the damaged integrity of the BBB. We subsequently demonstrated the PCN033-induced downregulation of the TJ proteins *in vivo* and *in vitro*, as well as the disorganization of junctional proteins around the brain vessels. This downregulation and remodeling of TJ proteins further explain the bacterial induced increase of the BBB permeability.

Multiple factors have been reported to be involved in regulation of the permeability, and a prominent regulator involving is the VEGFA. In CNS inflammation, VEGFA and TYMP worked together for decreasing Claudin-5 and Occludin [39]. Moreover, several lines of evidence have supported the astrocyte-derived VEGFA as an important inducer of BBB disruption via endothelial nitric oxide synthase (eNOS)-mediated downregulation of Claudin-5 and Occludin [40, 41]. Despite these advances, little is known concerning VEGFA, as well as the involving mechanism, in meningitic *E. coli* induced BBB disruption. In our study, we found the endothelial-derived VEGFA also contributes to the BBB disruption, by the demonstrations that meningitic *E. coli* PCN033 infection induced the expression of VEGFA *in vitro* and *in vivo*, and exogenous VEGFA dose-dependently increased the BBB permeability, while blocking VEGFA function largely blocked the BBB disruption. Moreover, blocking of the VEGFA signaling by VEGFR inhibitor significantly restored the PCN033-mediated downregulation of the TJ proteins. However, this treatment did not fully restore the TJ proteins downregulation, especially the downregulation of Claudin-5, suggesting that other molecules, except for VEGFA, may also mediate the decreased expression of TJ proteins by meningitic *E. coli*. In contrast, we observed a much lower induction of VEGFA mRNA as well as the protein, in hBMEC upon HB101 infection, compared to that of PCN033 infection (Supplementary Figure S4A and S4B). Thus, our observations support the essential role of VEGFA in regulating BBB permeability in response to meningitic *E. coli*.

In addition, we observed the important role of Snail-1 in regulation of the TJ proteins upon meningitic *E. coli* challenge. Increasing studies have supported the implication of Snail-1 in cell junctions. The over expression of Snail-1 can destroy the top complex of vascular endothelial cells [42]. Snail-1 can downregulate the expression of Claudin-4, Claudin-5 and Claudin-7, in which Claudin-5 played the directly role in maintaining the close connection of cells [43]. Moreover, Snail-1 downregulated the Occludin transcription by binding to a special E-box in the Occludin promoter [44]. Recently, Snail-1 was reported to be necessary and sufficient in the disruption of the TJ proteins in GBS meningitis [26]. In our study, we similarly demonstrated the induction of Snail-1 by PCN033, but not by HB101 (Supplementary Figure S4C), as well as the negative regulation of Snail-1 on those junctional proteins including ZO-1, Occludin, Claudin-5, and β-catenin. Although the decreased expression of TJ proteins were not fully restored by Snail-1 knocking-down, this reflected to some extent a negative effect of Snail-1 on the TJ proteins. More factors involving in this process are to be identified to better understand the regulation of the BBB by meningitic *E. coli*. But based on current observations, we could at least proposed that two host factors, VEGFA and Snail-1, play the negatively regulatory roles on the TJ proteins and thus mediate the BBB disruption.

In addition to specific host molecules that participate in the TJ proteins regulation, another factor that driving the BBB disruption is the infection-induced inflammation. Actually, multiple studies supported that BBB disruption has been intrinsically linked to the action of proinflammatory cytokines [45, 46]. For example, the co-involvement of TNF-α and IL-6 during BBB injury has been reported in various models of neuroinflammation [47, 48]. IL-1β may lead to the damage of the BBB by inducing the overexpression of VEGFA [49]. In our study, we demonstrated the significant induction of cytokines and chemokines in response to meningitic *E. coli* infection *in vivo*, and the time-dependent increase of proinflammatory cytokines and chemokines in hBMEC. We thus speculate that meningitic *E. coli* primarily induces the proinflammatory cytokines and chemokines in hBMEC. The activated hBMEC, as well as the released cytokines and chemokines, recruit more circulating inflammatory cells and finally result in neuroinflammation storm. More importantly, we found the significant induction of VEGFA and Snail-1 in hBMEC by the treatment of cytokines (IL-6, IL-1β, and TNF-α). This prompts us to believe that the proinflammatory factors, especially IL-6, IL-1β, and TNF-α, derived from the brain or the periphery, could promote the disruption of BBB, probably via upregulating the transcription of VEGFA and Snail-1.

Meningitic *E. coli* induction of the BBB disruption is a complicated interaction between bacteria and the host which requires the specific signaling transduction. Here, our results demonstrated that bacterial induction of VEGFA and Snail-1 largely depends on the activation of MAPK-ERK1/2. The MAPK-ERK1/2 signaling pathway has been demonstrated to modulate the expression of TJ proteins and alter the molecular composition within TJ proteins complexes [50], e.g. activation of ERK1/2 leads to increased ZO-1 degradation during traumatic brain injury [51], decreased ZO-1 and Claudin-5 expression in brain endothelium during HIV infection [52, 53], and reduced Occludin and Claudin-5 expression in BBB endothelium during ischemic stroke [54]. Further, we observed that TLR2 agonist Pam3CSK4 dose-dependently contributed to the activation of ERK1/2, as well as upregulation of VEGFA and Snail-1, suggesting the involvement of meningitic *E. coli* induced TLR2-MAPK-ERK1/2 signaling cascade in mediating the induction of VEGFA and Snail-1. Meanwhile, this signaling cascade in hBMEC might be the molecular basis for the meningitic *E. coli*-induced generation of the cytokines and chemokines, which were also the important contributor in VEGFA and Snail-1 induction. Together, these observations lead to the hypothesis that VEGFA and Snail-1 are the essential intermedium docks that mediate the regulation of TLR2-MAPK-ERK1/2 on the TJ proteins.

**Figure 7:**
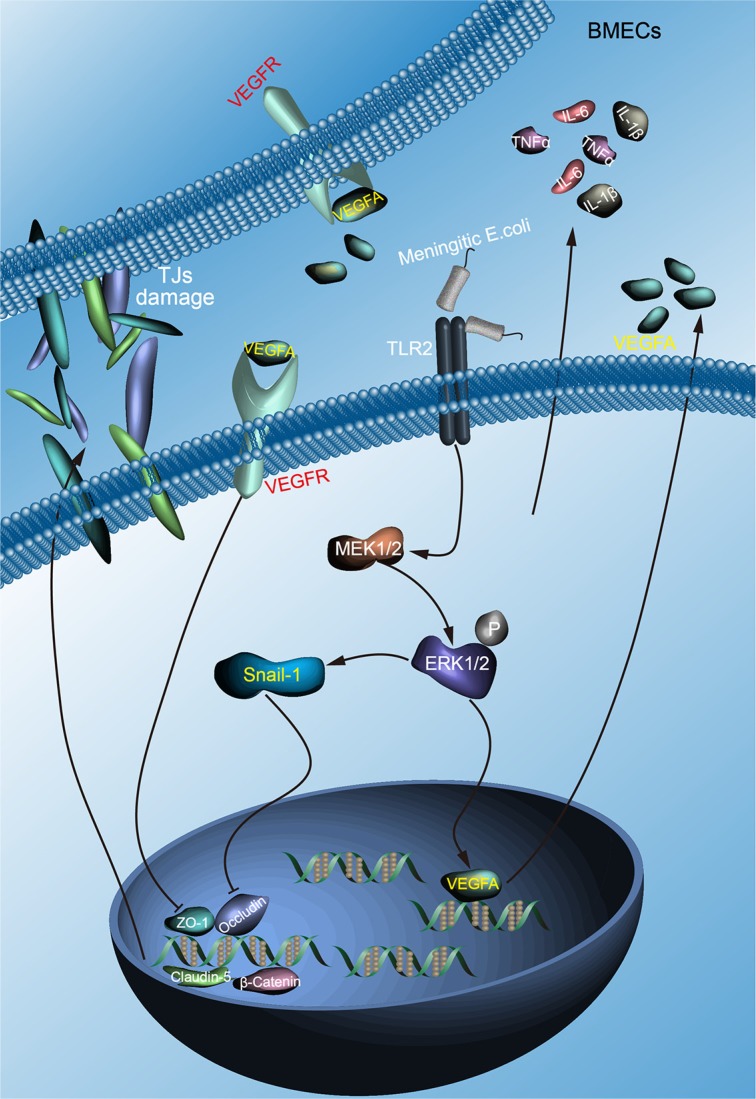
Schematic presentation of the importance of VEGFA and Snail-1 in meningitic *E. coli* induced disruption of the BBB Meningitic *E. coli* invasion of the BMECs triggers the activation of TLR2-MAPK-ERK1/2 signaling cascade, leading to the upregulation of VEGFA and Snail-1. On one hand, VEGFA secretion and action on its receptor VEGFR negatively regulate the transcription of TJ proteins; on the other hand, induction of Snail-1 could also directly negatively regulate the TJ proteins, both resulting in the disruption of the BBB. In addition, the infection-induced production of multiple cytokines and chemokines effectively conduced to the upregulation of VEGFA and Snail-1, further leading to the damage of the BBB integrity.

In summary, we highlight the importance of VEGFA and Snail-1 in meningitic *E. coli* induced disruption of the BBB (Figure 7). We demonstrate that meningitic *E. coli* PCN033 enhances the BBB permeability through downregulating as well as remodeling the TJ proteins. We further confirm the induction of VEGFA and Snail-1 by meningitic *E. coli* and their negative regulation of the TJ proteins via TLR2-MAPK-ERK1/2 signaling cascade. Moreover, meningitic *E. coli*-induced high level of cytokines and chemokines are also important contributors in the infection-induced disruption of the BBB, via directly upregulating the VEGFA and Snail-1 in BMECs. Finding the key molecules that are regulative to the TJ proteins in bacterial meningitis is of great significance, and our current findings robustly support the contribution of VEGFA and Snail-1 in meningitic *E. coli*-induced BBB disruption, and therefore represent the important targets in the prevention and therapy of CNS disorders.

## MATERIALS AND METHODS

### Bacterial strains and cell culture

All ExPEC strains used in this study were isolated from extraintestinal tissues, among which PCN033 was a swine cerebrospinal fluid isolate [27]. *E. coli* strain RS218 (O18:K1:H7) was obtained also as a cerebrospinal fluid isolate from a case of neonate meningitis, which used as the positive control strain, and *E. coli* K12 strain HB101 was as the negative strain [13, 55]. All *E. coli* strains were grown aerobically at 37°C in Luria–Bertani (LB) medium unless other specified.

The hBMEC cell line was kindly provided by Prof. Kwang Sik Kim in Johns Hopkins University School of Medicine [56, 57], and was routinely cultured in RPMI1640 supplemented with 10% heat-inactivated fetal bovine serum, 10% Nu-Serum, 2 mM L-glutamine, 1 mM Sodium pyruvate, nonessential amino acids, vitamins, and penicillin and streptomycin (100 U/mL) in 37°C incubator under 5% CO_2_ until monolayer confluence. In some experiments, confluent hBMEC was washed thrice with Hanks' Balanced Salt Solution (Corning Cellgro, Manassas, VA, USA) and starved in serum-free medium (1:1 mixture of Ham's F-12 and M-199) for 16–18 h before further treatment. As specified in some assays, cells were pretreated with various inhibitors prior to addition of the bacteria.

### Reagents, antibodies and shRNA plasmid

Evan's Blue dye and NaF were purchased from Santa Cruz Biotechnology (Santa Cruz, CA, USA). Motesanib diphosphate, ZM323881, and SAR131675 were from MedChemexpress (Monmouth, NJ, USA). U0126, SB202190 and SP600125 were from Cayman Chemical Company (Ann Arbor, MI, USA). Pam3CSK4 were purchased from InvivoGen (San Diego, CA, USA). Recombinant Human IL-1β, IL-6 and TNF-α were from Novoprotein (Summit, NJ, USA). Human VEGFA ELISA Kit and Mouse VEGFA ELISA Kit were obtained from 4A Biotech, Co., Ltd (Beijing, China). For the Western Blotting, anti-Claudin-5 (rabbit) and anti-Occludin (rabbit) antibodies were from Aviva Systems Biology (San Diego, CA, USA). The β- catenin (rabbit) and β-actin (mouse) antibodies were from HuaAn Biotechnology Co., Ltd (Hangzhou, China). ZO-1, ERK1/2 and phospho-ERK1/2 (all rabbit) antibodies were from Cell Signaling (Danvers, MA, USA). For the IHC, anti-β-catenin and anti-ZO-1 antibodies (all rabbit) were from HuaAn Biotechnology. Occludin and Claudin-5 antibodies (all rabbit) were from Arigo Biolaboratories (Hsinchu, Taiwan). For the IF, anti-CD34 (mouse), anti-ZO-1 and anti-Occludin antibodies (all rabbit) were from Proteintech (Chicago, IL, USA). The β-catenin (rabbit) antibody was from Bioss (Woburn, MA, USA) and the anti-Claudin-5 (rabbit) was from Abcam (Cambridge, MA, USA). FITC-labeled Goat anti-mouse and Cy3-labeled Goat anti-rabbit antibodies were from Beyotime Institute of Biotechnology (China). The Lipofectamine 3000 transfection reagent was purchased from Invitrogen (Carlsbad, CA, USA). The Snail-1 shRNA plasmid and control shRNA plasmid-A were obtained from Santa Cruz Biotechnology.

### *In vitro* invasion and *in vivo* colonization

The *in vitro* ability of *E. coli* strains invasion of hBMEC was determined according to the previous methods [58]. Briefly, overnight cultured *E. coli* strains were resuspended in experimental medium (M199-Ham F12 [1:1] medium containing 5% heat-inactivated FBS) and added into the confluent hBMEC monolayer grown in 24-well plate at multiplicity of infection (MOI) of 10 (approximately 10^7^CFU/well) to allow invading at 37°C for 90 min. Cells were then washed to remove the free bacteria and incubated in medium containing specific antibiotics (based on our early MIC tests for each strain) for another 1 h to kill extracellular bacteria. Finally, cells were extensively washed and lysed in 0.025% Triton X-100 buffer. The released intracellular bacteria were quantified by appropriate dilutions and plating. Results were calculated as percentages of the initial inoculums, and presented as percent relative invasion compared to that of the positive strain RS218. Each invasion assay was performed in triplicate.

For the *in vivo* colonization assay, the SPF female KM mice at 4 weeks of age, purchased from Center for Disease Control in Hubei province of China (Quality Certificate No.42000600007603), were used for induction of hematogenous bacterial meningitis. Mice were injected intravenously with 100 μL bacterial suspension containing 1 × 10^7^ CFUs that diluted in phosphate-buffered saline (PBS, pH7.4). At each time point post-inoculation, the mice were anesthetized and blood was collected for quantitative circulating bacterial cultures. Mice were subsequently perfused as previously described [13], and the brains, spleens and kidneys were homogenized and plated to determine the bacterial counts.

### *In vivo* BBB permeability measurement

BBB permeability was assessed either by using the Evan's blue dye (961 Da), which binds to serum albumin to become a protein tracer with high-molecular-weight *in vivo*, or by using the sodium fluorescein dye (NaF, 376 Da), which remains an unbound small molecule in the circulation [59]. Briefly, mice were challenged as mentioned above. 500 μL Evan's blue (5 mg/ml) was injected via the tail vein and allowed to circulate for 10 min, at which time mice were sacrificed and perfused transcardially with sterile PBS. Brains were then removed and photographed for the staining of the dye. Likewise, NaF was also utilized as a tracer molecule to evaluate the BBB permeability. Mice were injected with 10 mg of NaF dissolved in 100 μL sterile saline following challenge. After 10 min diffusion of the NaF, peripheral blood and the brains were collected for the fluorescence reading as described previously [59]. As specified in some experiments, recombinant VEGFA (VEGF165) was injected intravenously at the increasing dosage (1 ng, 10 ng, 50 ng), and motesanib diphosphate (25 mg/kg, applied at the therapeutic dosage) was intraperitoneally administrated 2 h before bacterial challenge.

### Western blotting

Challenged hBMEC and mice brains were lysed in RIPA buffer with protease inhibitor cocktail (Sigma-Aldrich, USA), sonicated and centrifuged at 10,000 g for 10 min at 4°C. The insoluble debris was removed and protein concentration in supernatant was measured using BCA protein assay kit (Beyotime, China). Brain extracts or cell lysates were then separated on 10%–12% sodium dodecyl sulfate polyacrylamide gel electrophoresis (SDS-PAGE), and transferred to polyvinylidene difluoride (PVDF) membranes (Bio-Rad, CA). The blots were blocked in 5% BSA in Tris-buffered saline with Tween 20 (TBST) for 2 h at room temperature, and then incubated overnight with either one of anti-ZO-1 (Abcam, USA), Claudin-5 (AVIVA, USA), Occludin (AVIVA, USA), β-Catenin (HuaAn, China), β-actin (HuaAn, China), ERK1/2 (CST, USA), p-ERK1/2 (CST, USA) antibodies. After washing, the blots were incubated with species-specific horseradish peroxidase-conjugated antibodies and finally visualized with the ECL reagents (Bio-rad, USA). All Western Blots were densitometrically quantified by using the ImageJ software, and the results were analyzed as the relative immunoreactivity of each protein normalized to the respective loading control.

### RNA isolation and quantitative real-time PCR analysis

Total RNAs from brain lysates or cells were extracted using the TRIzol reagent (Invitrogen, Carlsbad, CA, USA). Contaminating genomic DNA was removed by DNase I treatment (New England Biolabs, Ipswich, MA, USA). Aliquots (1 μg) of the total RNA in each sample were subjected to cDNA synthesis using PrimeScript^TM^ RT reagent Kit with gDNA Eraser (Takara, Japan). Real-time PCR was performed with a ViiA^TM^ 7 Real-Time PCR System (Applied BioSystems, Foster City, CA, USA) using Power SYBR Green PCR master mix (Applied BioSystems), according to the manufacturers' instructions. Primers for the quantitation real-time PCR were listed in Supplementary Table S1. The amplification conditions were: 50°C for 2 min and 95°C for 10 min, followed by 40 cycles of 95°C for 15s and 60°C for 1 min. The products were then applied to a melt curve stage with denaturation at 95°C for 15s, anneal at 60°C for 1 min, and slow dissociation by ramping from 60°C to 95°C at 0.05°C/s to ensure the specificity of the PCR products. Expression levels of the target genes were normalized to either β-actin or GAPDH. Each assay was performed in triplicate.

### Histopathological examinations, IHC and IF analysis

Mice showing the typical CNS disorder post infection were anesthetized with ketamine-xylazine (0.1 mL/10g) and perfused with PBS. For the swine samples, 4–5 weeks healthy pigs were challenged with 8 × 10^8^ CFU PCN033 or PBS via ear vein for seven days. The brain samples were collected, fixed in 4% formaldehyde solution followed by embedding in paraffin. Individual 6 μm sections were mounted on adhesive glass slides, dewaxed in xylene, and rehydrated in descending graded ethanol for the hematoxylin and eosin (H&E) histopathological staining [60].

For IHC, the paraffin sections were deparaffinized and rehydrated in xylene and ethanol. Endogenous peroxidase was quenched by incubation in 3% hydrogen peroxide, and antigen retrieval was performed in 10 mM citrate buffer. Sections were then were blocked with 5% BSA in PBS for 1 h at room temperature, followed by incubation with diluted primary antibody at 4°C overnight. After washing with PBS, secondary antibodies were applied and Diaminobenzidine (DAB) was utilized for color development [59]. Sections were photographed and analyzed using BX41 Microscope (Olympus, Tokyo, Japan). For IF, the immunostaining were performed as published [61].

### Secretory VEGFA determination by ELISA

For *in vitro* determination of secretory VEGFA, cells were seeded at 1 × 10^5^ in the 24-well plate and cultured until confluence. Cells were then serum-starved and stimulated with meningitic *E. coli* PCN033 at a MOI of 10 for specified periods of time, and the culture supernatant in each well was collected. For *in vivo* determination, mice were challenged with the bacteria, and the serum and the brains were collected as mentioned above. The secretory VEGFA from the culture supernatants, serum, as well as the brains were quantified using the VEGFA ELISA Kit, purchased from 4A Biotech (Beijing, China), following the procedures provided by the manufacturer.

### Transfection

The hBMEC was cultured until 50% to 60% confluence in 6-well plate and transfected with either the shRNA targeting Snail-1 (Santa Cruz, USA) or the control shRNA plasmid (Santa Cruz, USA) using the Lipofectamine 3000 transfection reagent (Invitrogen, Carlsbad, CA, USA) according to manufacturers' instructions. Briefly, liquid A (5 μg plasmid, 10 μL P3000 and 250 μL opti-MEM) and liquid B (7.5 μL Lipo3000 and 250 μL opti-MEM) were gently mixed and incubated at room temperature for 5 min. This mixed suspension was then added dropwise to the serum-starved cells and incubated at 37°C incubator with 5% CO_2_ for 4–6 h. The medium containing puromycin (100 μg/ml) was added and maintained for 2–3 weeks, and antibiotic-resistant colonies were pooled or separated into individual clones for following analysis.

### Multiplex cytokine and chemokine assays

Mice were challenged with the meningitic *E. coli* PCN033 and sacrificed at the indicated time points. Serum was prepared from the blood samples and stored at −80°C until processing. The right hemisphere of each mouse was lysed in the RIPA buffer with protease inhibitor cocktail and then centrifuged for 10 min at 12,000 rpm to remove debris. Protein concentrations of the brain lysates were adjusted with PBS buffer to 10 mg/mL and stored at −80°C. The serum and brain extracts were applied for the measurement of 26 preselected cytokines and chemokines using Affymetrix ProcartaPlex Mouse Cytokine & Chemokine Panel 1 (26 plex) according to the manufacturer's instructions (Fremont, CA, USA). Cytokines and chemokines measured were as follows: IL-1β, IL-2, IL-4, IL-5, IL-6, IL-9, IL-10, IL-12p70, IL-13, IL-17A, IL-18, IL-22, IL-23, IL-27, TNF-α, IFN-γ, granulocyte-monocyte colony stimulating factor (GM-CSF), monocyte chemoattractive protein (MCP)-1 (CCL2), MCP-3 (CCL7), CCL5 (RANTES), macrophage inflammatory protein (MIP)-1α (CCL3), MIP-1β (CCL4), MIP-2 (CCL8), IFN-γ induced protein 10 (IP10/CXCL10), CXCL1 (GROα) and Eotaxin (CCL11). Whole procedures of the multiplex cytokine determination followed the previous descriptions [62]. Concentrated mouse recombinant cytokines and chemokines were provided in the kit to establish standard curves and maximize the sensitivity and the assay dynamic range. Cytokine and chemokine levels were determined using a multiplex array reader from Luminex™ Instrumentation System (BioPlex Workstation from Bio-Rad Laboratories). The concentration was calculated using Bio-Plex Manager Software provided by the manufacturer (Bio-Rad Laboratories, Hercules, CA).

### Gene expression profiling using quantiGene plex 2.0 reagent system

Right hemispheres of the brain, as mentioned above, were proceeded for the protein level determination, and the remaining left hemispheres were collected for the expression profiling of those cytokines and chemokines above listed. Briefly, QuantiGene sample processing kit (eBioscience, San Diego, CA, USA) was used to homogenize the brains. RNAs from different brains were captured by fluorescent microspheres and subsequently quantified by the approach of Affymetrix QuantiGene Plex 2.0 Reagent system according to the manufacturer's instructions (Fremont, CA, USA) [63]. Signals of cascade amplification were detected by Bio-Plex 100 × MAP technology and analyzed using Bio-Plex 6.0 software (Bio-Rad Laboratories, Hercules, CA). Mean fluorescence intensity (MFI) signals generated from each bead were proportional to the amount of each mRNA captured on the surface of each generated specific probe set [64]. The geometric means of two housekeeping genes (*Ppib*, NM_011149; *Tbp*, NM_013684) were used for normalization of each sample. Fold changes were presented as the relative ratios between normalized values of infected mice and that of the control mice.

### Statistical analysis

Data were expressed as mean ± standard deviation (mean ± SD) unless otherwise specified. Significance of the differences between each group was analyzed by one-way analysis of variance (ANOVA) embedded in GraphPad Prism, version 6.0. Differences of the bacterial counts in invasion assays *in vitro* were determined by Student's *t-test*, and differences of bacterial colonization *in vivo* between each group of mice were determined by the Wilcoxon rank-sum test. *P* < 0.05 (*) was considered significant, and *p* < 0.01 (**), as well as < 0.001 (***) were all considered extremely significant.

### Ethics statement

The current study was carried out in strict accordance with the guidelines established by the China Regulations for the Administration of Affairs Concerning Experimental Animals (1988) and Regulations for the Administration of Affairs Concerning Experimental Animals in Hubei province (2005) (Project No.00133493 and Animal Welfare Assurance No.150402). All procedures and handling techniques were approved by the Committee for Protection, Supervision and Control of Experiments on Animals guidelines, Huazhong Agriculture University (Permit No.SYXK2015-0084). All efforts were made to provide the ethical treatment and minimize suffering of animals employed in this study.

## SUPPLEMENTARY MATERIALS FIGURES AND TABLE


