# Supplementary material for: Induction of VEGFA and Snail-1 by meningitic Escherichia coli mediates disruption of the blood-brain barrier
Source: Oncotarget. 2016 Aug 30;7(39):63839–55. doi: 10.18632/oncotarget.11696 (PMC5325408; doi:10.18632/oncotarget.11696)
Supplement: Supplementary file 1 [file oncotarget-07-63839-s001.pdf]

Induction of VEGFA and Snail-1 by meningitic *Escherichia coli* mediates disruption of the blood-brain barrier

Supplementary Materials

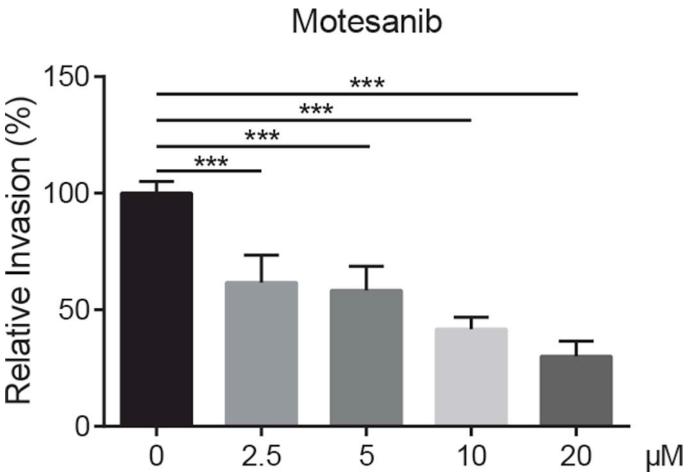

Supplementary Figure S1: The inhibition of VEGFR on meningitic *E. coli* invasion of the hBMEC. VEGFR inhibitor motesanib significantly decreased meningitic *E. coli* PCN033 invasion of hBMEC in a dose-dependent manner.

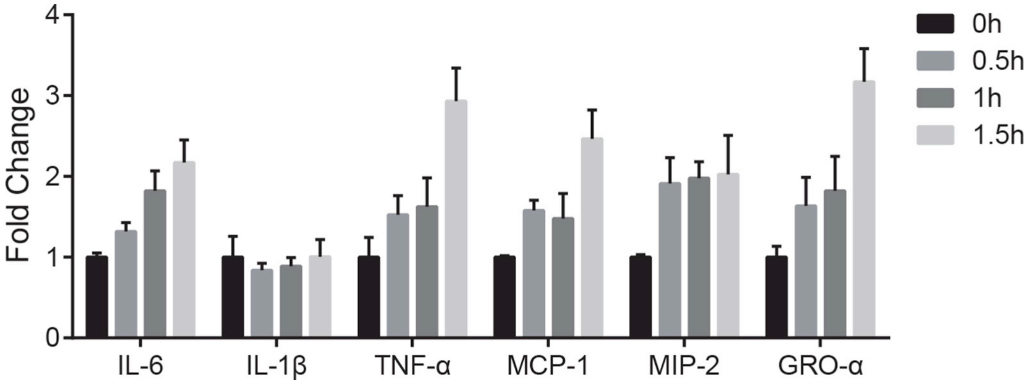

Supplementary Figure S2: The induction of proinflammatory factors by non-meningitic *E. coli* strain HB101. The transcriptional changes of cytokines and chemokines in hBMEC upon HB101 infection.

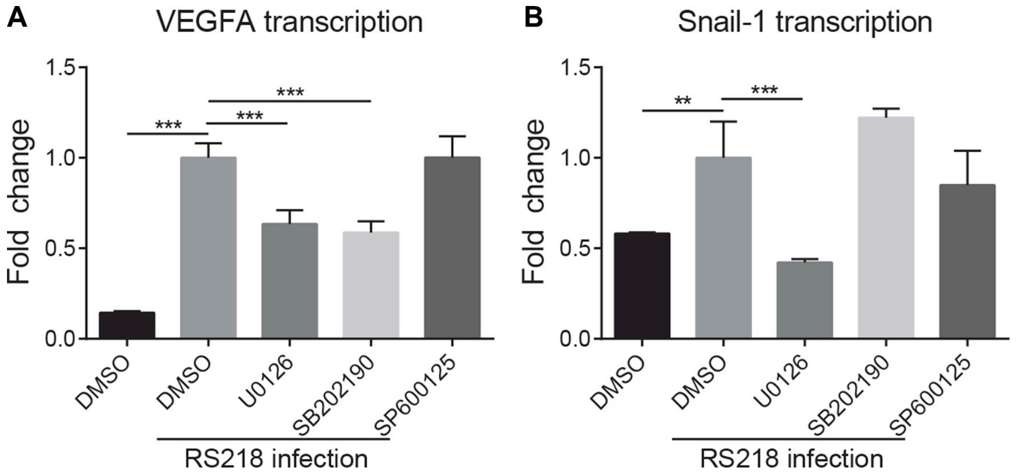

Supplementary Figure S3: *E. coli* K1 strain RS218 induction of VEGFA and Snail-1 requires the activation of MAPK-ERK1/2 signaling. Effects of the MAPK signaling inhibitors on the *E. coli* K1 strain RS218-induced upregulation of VEGFA and Snail-1. (**A**) The upregulation of VEGFA could be significantly inhibited by the treatment of U0126 (selective inhibitor of ERK1/2) and SB202190 (selective inhibitor of p38). (**B**) The upregulation of Snail-1 could be significantly blocked by U0126. Both upregulation of VEGFA and Snail-1 were not affected by the treatment of SP600125 (specific inhibitor of JNK).

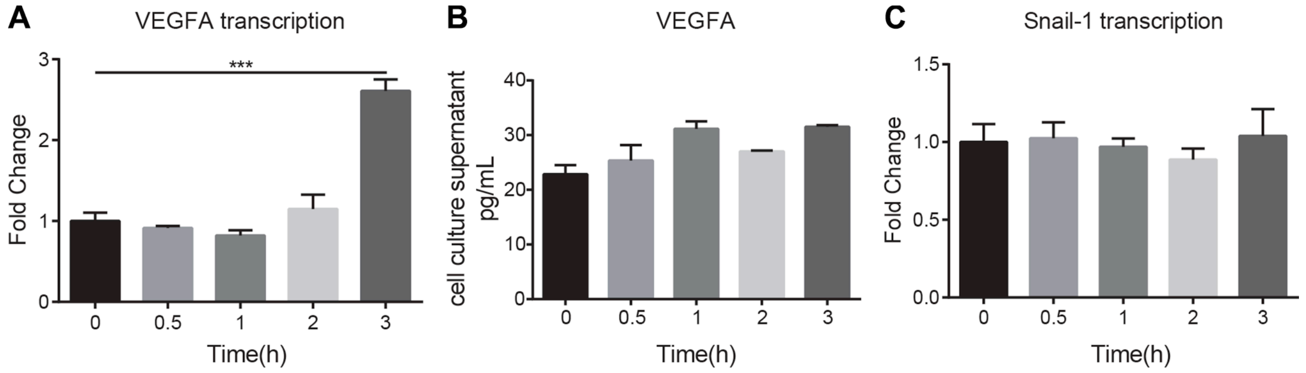

Supplementary Figure S4: Regulation of VEGFA and Snail-1 by non-meningitic *E. coli* strain HB101. (**A**) Real-time PCR analysis of the VEGFA transcription in hBMEC in response to HB101 infection at the MOI of 10. GAPDH was used as the internal reference for the normalization. (**B**) Determination of the secretory VEGFA in the hBMEC culture supernatant in response to HB101 by ELISA. (**C**) Real-time PCR analysis of Snail-1 transcription in hBMEC in response to HB101 challenge.

Supplementary Table S1: Primers used for the real-time PCR in this study

Primers	Nucleotide sequence (5′-3′)	Amplified fragments
P1	TGTGGAAGAGGATGAAGATGAAGA	Human ZO-1
P2	GGTGGAAGGATGCTGTTGTC	
P3	ACAAGCCACAAGATTACAAG	Human β-catenin
P4	ATCAGCAGTCTCATTCCAA	
P5	TTAACTTCGCCTGTGGAT	Human Occludin
P6	TGTGTAGTCTGTCTCATAGTG	
P7	CGCCTTCCTGGACCACAACAT	Human Claudin-5
P8	CCAGCACCGAGTCGTACACTT	
P9	TGCCTTGCTGCTCTACCT	Human VEGFA
P10	GACATCCATGAACTTCACCACTT	
P11	ACTCGGATGTGAAGAGATAC	Human Snail-1
P12	AGACTCTTGGTGCTTGTG	
P13	CCTTCGGTCCAGTTGCCTTCT	Human IL-6
P14	GAGGTGAGTGGCTGTCTGTGT	
P15	AGGATATGGAGCAACAAGT	Human IL-1β
P16	GCAGGACAGGTACAGATT	
P17	AATGGCGTGGAGCTGAGA	Human TNF-α
P18	TGGCAGAGAGGAGGTTGAC	
P19	ATAGCAGCCACCTTCATT	Human MCP-1
P20	GCTTCTTTGGGACACTTG	
P21	AGTGTGAAGGTGAAGTCC	Human MIP-2
P22	CTTTCTGCCCATTCTTGAG	
P23	TGCTGCTCCTGCTCCTGGTA	Human GRO-α
P24	TGTGGCTATGACTTCGGTTTGG	
P25	TGCCTCCTGCACCACCAACT	Human GAPDH
P26	CGCCTGCTTCACCACCTTC	
P27	GTCCCTCCTCTGATACCTTCCTC	Mouse ZO-1
P28	CTGGCAGTGTCATTCACATCTTTC	
P29	AGCCACAGGATTACAAGAA	Mouse β-catenin
P30	CCAATGTCCAGTCCAAGA	
P31	ATGGAGGCTATGGCTATGG	Mouse Occludin
P32	GGAAGCGATGAAGCAGAAG	
P33	AAAGGGCTCATTGCGGTGGTTGT	Mouse Claudin-5
P34	CTCGCTGTTGGAGTTCAGAAGTGGA	
P35	CCATTCTCCTGCTCCCACT	Mouse Snail-1
P36	TGGCACTGGTATCTCTTCACA	
P37	CACTGCCGCATCCTCTTCCTCCC	Mouse β-actin
P38	CAATAGTGATGACCTGGCCGT	
